# The Evidence of Physiotherapy Scoliosis-Specific Exercises in Treating Secondary Postural Deviations in Mild and Moderate Idiopathic Scoliosis: A Scoping Review

**DOI:** 10.3390/jfmk11030288

**Published:** 2026-07-24

**Authors:** Shkurta Rrecaj Malaj, Mejdi Aliu, Ardiana Murtezani, Tine Kovačič

**Affiliations:** 1 Physiotherapy Department, Faculty of Medicine, University of Prishtina, 10000 Prishtina, Kosovo; shkurta.malaj@uni-pr.edu (S.R.M.); mejdi.aliu@uni-pr.edu (M.A.); 2 Physiotherapy Department, Alma Mater Europaea University, 2000 Maribor, Slovenia; tine.kovacic@almamater.si

**Keywords:** idiopathic scoliosis, physiotherapy scoliosis-specific exercises, pelvic asymmetry, trunk symmetry and morphology, spinal mobility, kyphosis, lordosis

## Abstract

Idiopathic scoliosis (IS) is a three-dimensional spinal deformity often associated with secondary postural deviations (pelvic asymmetry, altered kyphosis/lordosis, and postural imbalance), which may negatively affect psychological well-being and quality of life (QoL). Physiotherapy interventions designed specifically for scoliosis, known as Physiotherapy scoliosis-specific exercises (PSSE), have shown effectiveness in improving primary structural outcomes such as Cobb angle (CA) and angle of trunk rotation (ATR); however, their influence on secondary postural deviations is still unclear. This scoping review aimed to summarize current evidence of PSSE on secondary postural deviations in children, adolescents, and young adults with mild to moderate IS: Following PRISMA-ScR guidelines, searches were conducted in PEDro, PubMed, Scopus and Google Scholar for studies published in the last 10 years. Eligible studies were appraised using study design-specific quality assessment tools. Randomized controlled trials (RCT) were evaluated using the Physiotherapy Evidence Database Scale (PEDro), the retrospective cohort study was assessed using the Newcastle–Ottawa Scale (NOS), and the prospective observational study was evaluated using the Joanna Briggs Institute (JBI) Critical Appraisal Checklist. Outcomes included pelvic asymmetry, spinal deviations, mobility, trunk symmetry/morphology, balance, proprioception, sensorimotor control, and QoL. Six studies involving 230 participants with mild-to-moderate IS were included. PSSE was associated with improvements in CA, ATR, postural alignment, spinal mobility, and QoL. Adjunctive interventions, including manual therapy (MT), proprioceptive neuromuscular facilitation (PNF), and hippotherapy, generally produced greater improvements in postural symmetry, pelvic alignment, proprioception, and sensorimotor control than PSSE alone. PSSE appears to improve structural, postural, and functional outcomes in individuals with IS, with adjunctive interventions potentially enhancing these effects. However, current evidence is limited by heterogeneity, highlighting the need for high-quality RCTs with standardized protocols and long-term follow-up.

## 1. Introduction

Idiopathic scoliosis (IS) is a complex three-dimensional deformity of the spine with an unknown etiology [1,2,3]. This scoliotic deformity is characterized by a lateral deviation of the spine, which is measured by the Cobb angle (CA) [4,5]. It is also accompanied by vertebral rotation, which becomes clinically evident through the Angle of Trunk Rotation (ATR) assessed during Adam’s forward bend test [5,6]. These structural changes constitute the primary structural deformity or primary deviations. They represent the fundamental and fixed deformity of the spinal column.

Progression of the curve during growth can result in cosmetic deformity, pain, and long-term functional impairment. Because of these structural deviations, postural imbalance, spinal muscle weakness, and, in some cases, chronic pain may also occur [7,8,9,10]. The structural spinal changes may prompt the body to develop a series of compensatory or adaptive adjustments to preserve postural equilibrium and maintain an upright standing position. These adaptations may include pelvic asymmetry, trunk imbalance (frontal imbalance), trunk inclination (forward or backward), alterations in sagittal spinal curvatures (hyperkyphosis or hyperlordosis), shoulder asymmetry, and head or neck deviation. These features are commonly considered secondary postural deviations or asymmetries and are often interpreted as compensatory adaptations to the primary structural deformity. However, current evidence suggests that neuromuscular and sensorimotor alterations may also contribute to the development and progression of IS. Therefore, secondary postural deviations likely reflect a combination of compensatory adaptations and underlying neuromuscular mechanisms rather than being solely consequences of the primary structural deformity [11]. Secondary postural deviations can further affect psychological well-being and quality of life (QoL). Poor postural aesthetics may diminish self-esteem, limit social participation, and interfere with daily functioning [1,9,12,13,14].

IS affects both the structure and the function of the spine and pelvis [15]. Early and effective management is therefore essential. Conservative approaches represent the first-line treatment for mild to moderate IS cases. These include bracing and Physiotherapy Scoliosis-Specific Exercises (PSSE), both of which aim to prevent further progression of spinal deformity [16,17,18]. Scoliosis Orthopaedic and Rehabilitation Treatment (SOSORT) uses the term PSSE to encompass all exercise schools developed for treating scoliosis. Recent research has demonstrated the effectiveness of PSSE in managing children and adolescents with IS [19]. Multiple standardized exercise approaches, including the Scientific Exercise Approach to Scoliosis (SEAS), Schroth exercise (SE), Dobomed, Pilates, Side-Shift, Lyon, and Functional Individual Therapy for Scoliosis (FITS), are classified under PSSE. Their benefit in treating IS had been widely documented in the scientific literature [6,9,20,21,22,23,24,25,26,27,28]. PSSE should therefore be performed in accordance with the SOSORT Consensus. A comprehensive program includes 3D autocorrection exercises, postural correction and stabilization, training in activities of daily living (ADLs), and patient education [29,30,31]. PSSE has shown effectiveness in improving primary structural deviation parameters, particularly CA and ATR, and it has also shown positive effects on QoL [22,32,33,34].

Although the effectiveness of PSSE in improving primary structural deviation parameters and QoL is well established, its influence on secondary postural deviations remains insufficiently explored. To the best of our knowledge, our literature search identified only a limited number of studies that specifically investigated the effects of PSSE on secondary postural deviations or compared multimodal PSSE-based interventions targeting these parameters. Furthermore, no previous review was found that systematically evaluated the evidence of PSSE on secondary postural deviations. This scoping review addresses this gap by synthesizing the existing evidence on the effects of PSSE on secondary postural deviations, including pelvic parameters (torsion, rotation, and tilt), trunk inclination and imbalance, and sagittal plane curvatures (kyphosis and lordosis), in children, adolescents, and young adults with mild to moderate IS. Accordingly, the aim of this review was to map and summarize the available evidence on these outcomes and to identify knowledge gaps that may inform future research.

## 2. Materials and Methods

**Figure 1:**
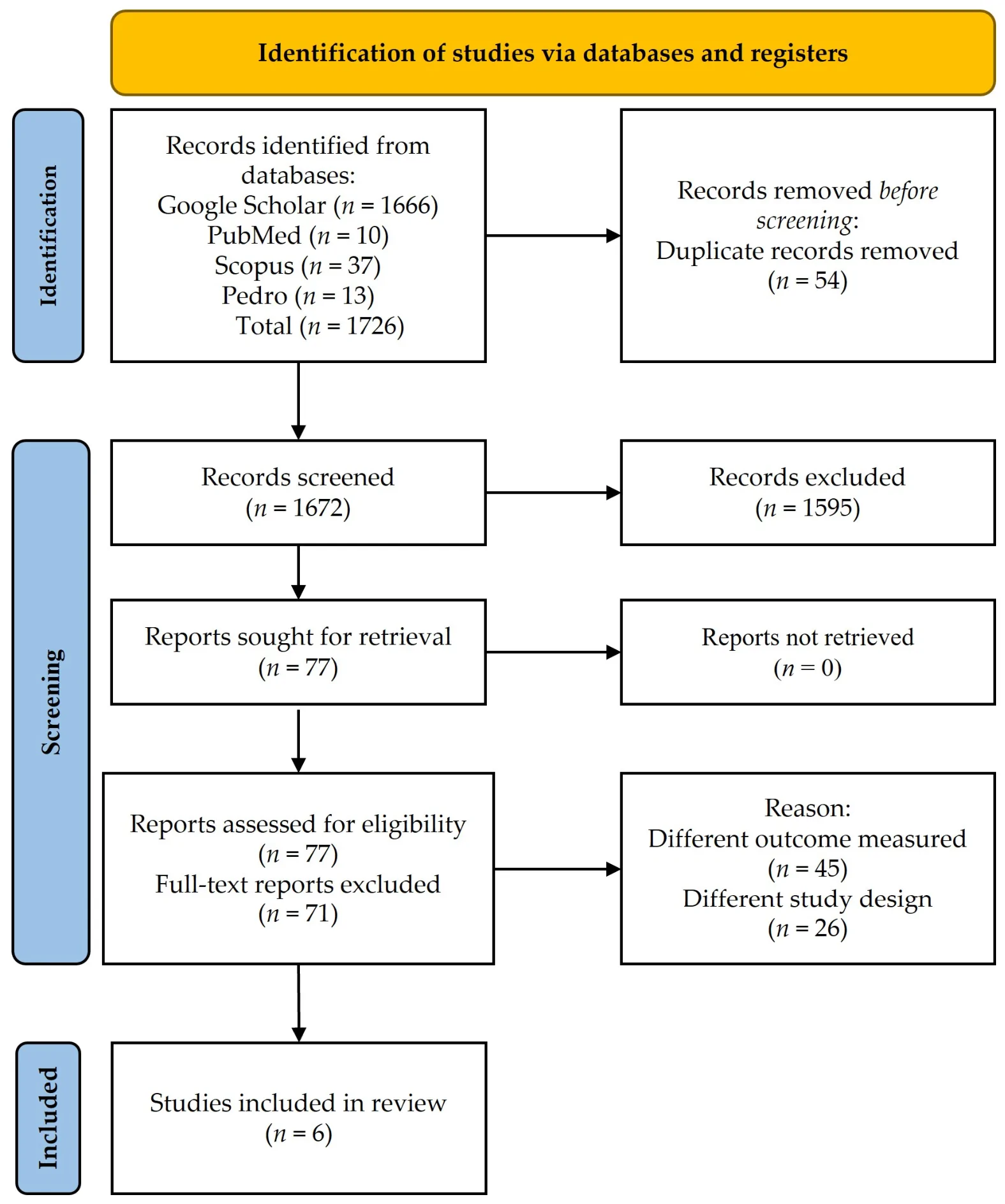
PRISMA-ScR flow diagram illustrating the identification, screening, eligibility assessment, and inclusion of studies in the scoping review.

This scoping review was conducted and reported in accordance with the methodological frameworks originally developed by Arksey and O’Malley [35] and later refined by Levac et al. [36], as well as the updated methodological guidance of the Joanna Briggs Institute (JBI) [37]. This scoping review was conducted and reported in accordance with the Preferred Reporting Items for Systematic Reviews and Meta-Analyses Extension for Scoping Reviews (PRISMA-ScR) statement. The study selection process is presented in the PRISMA-ScR flow diagram (Figure 1). The review protocol was prospectively registered in PROSPERO (CRD420251244609; registered on 3 December 2025).

This methodological approach is particularly well suited for mapping the existing literature on the effectiveness of PSSE as either a standalone intervention or in combination with other physiotherapeutic treatment methods, with a focus on both primary structural deviations and secondary postural deviations associated with IS. The methodology consisted of several key steps, including formulating the research question, conducting a comprehensive literature search, selecting relevant studies, extracting and organizing data, and synthesizing and presenting the findings.

### 2.1. Identifying the Research Question

As noted in the introduction, IS is often accompanied by secondary postural deviations that extend beyond the primary structural parameters (CA and ATR), including pelvic asymmetry, trunk asymmetry and imbalance, and sagittal plane curvatures such as kyphosis and lordosis, together with reduced spinal mobility, decreased balance, and impaired postural control. Clinical experience indicates that optimal therapeutic outcomes require improvements not only in radiographic correction but also in visible postural alignment. This is particularly relevant for the pelvis, trunk, and sagittal balance. Addressing secondary postural deviations remains essential because these deviations can significantly affect psychological well-being and overall QoL. With these considerations in mind, the following research question guided this scoping review: “What is the evidence of PSSE treatment on the correction of secondary postural deviations in children, adolescents, and young adults with mild to moderate IS?” This question establishes a clear aim and scope and ensures a comprehensive and systematic exploration of the existing literature.

### 2.2. Information Sources and Search Strategy

A comprehensive literature search was conducted using the Physiotherapy Evidence Database (PEDro), Scopus, PubMed, and Google Scholar. The search strategy and eligibility criteria were created through a structured approach that combined controlled vocabulary and keywords to capture all relevant studies. The following search terms were used: (“Adolescent Idiopathic Scoliosis” OR AIS OR “idiopathic scoliosis” OR “mild and moderate idiopathic scoliosis”) AND (“PSSE” OR “physiotherapy scoliosis-specific exercises” OR “physiotherapeutic exercises”) AND (“child” OR “adolescent” OR “adult”) AND (“pelvic asymmetry” OR “pelvic position”) AND (“trunk asymmetry” OR “trunk imbalance” OR “postural deviation”) AND (“posture correction” OR “asymmetry correction”) AND (“sagittal curvature” OR “secondary postural deviation”). The complete database-specific search strategies and retrieved results are detailed in Supplementary Material File S1.

### 2.3. Eligibility Criteria

The eligibility criteria were structured according to the Population, Concept, and Context (PCC) framework commonly used in reviews. The Population consisted of children, adolescents, and young adults diagnosed with mild to moderate IS [38]. The Concept consisted of PSSE used either as a standalone intervention or in combination with other physiotherapeutic methods. The Context required that studies report outcomes related to secondary postural deviations in addition to primary structural deviations of the spine. These criteria ensured the inclusion of studies relevant to the research question. Studies were included if they: (1) investigated the effects of PSSE, either as a standalone intervention, in combination with other physiotherapeutic methods, or in comparison with them, in children, adolescents, and young adults with mild to moderate IS; (2) reported outcomes not only on primary structural deviations of the spine (e.g., CA, ATR), and QoL but also on secondary postural deviations. At least one of the following parameters had to be reported: pelvic parameters (torsion, rotation, tilt), trunk inclination, trunk imbalance, or sagittal spinal deviations (e.g., kyphosis and lordosis), spinal mobility, trunk symmetry or morphology, balance, and proprioception; (3) were published in English; and (4) employed RCTs, clinical trials, or clinical studies as their study design. Studies were excluded if they: (1) were case reports, case series, review articles, letters to the editor, or conference abstracts; (2) were animal or in vitro studies; or (3) were published more than 10 years ago. The study selection proceeded through a three-stage screening approach, in which titles were screened first, followed by abstract screening, and finally full-text assessment of potentially eligible articles, following the method outlined by Khalil et al. [39]. All included studies were evaluated based on the pre-established inclusion and exclusion criteria. Two independent reviewers carried out the study selection and data extraction processes separately. Any discrepancies between the reviewers were resolved through discussion until consensus was reached.

### 2.4. Charting the Data

A data extraction form was developed to systematically collect and organize key information from each included study. The extracted data included the author (s), year of publication, study design, methodological quality, participant characteristics (age, sex, scoliosis severity), and sample size. It also captured the intervention dosage, including frequency, duration, and delivery format of PSSE. Additional extracted variables included the specific postural parameters evaluated (e.g., pelvic parameters, trunk inclination, trunk imbalance, and sagittal deviations, spinal mobility, trunk symmetry or morphology, balance, proprioception, outcomes related to primary structural deviations (e.g., CA and ATR), outcomes associated with secondary postural asymmetries, and the main findings regarding the effects of PSSE on both primary structural deviations and secondary postural deviations of the spine and body. The methodological quality of the included studies was assessed using study design-specific appraisal tools according to study design. Quasi-experimental studies were evaluated using the revised Joanna Briggs Institute (JBI) Critical Appraisal Checklist [40], retrospective cohort studies were assessed using the Newcastle–Ottawa Scale (NOS) [41], and RCTs were evaluated using the Physiotherapy Evidence Database (PEDro) Scale [42]. The database search was conducted between September and December 2025, and the final search was completed on 2 December 2025.

### 2.5. Collating, Summarizing, and Reporting the Results

In the final stage of the review process, the data extracted from the included studies were collated, synthesized, and summarized. The results were organized thematically to align with the research question. The organization focused on key outcome domains related to the effects of PSSE, both as a standalone intervention and in comparison with other physiotherapeutic methods, as well as when combined with other physiotherapeutic methods, on secondary postural deviations in children, adolescents, and young adults with mild to moderate IS. The main themes included at least one of the following parameters: pelvic parameters (torsion, rotation, tilt), trunk inclination and imbalance, sagittal plane deviations (e.g., kyphosis, lordosis), spinal mobility, trunk symmetry, trunk morphology, balance, and proprioception, combined changes in primary structural spinal deviation parameters (e.g., CA, ATR), and QoL when reported alongside postural outcomes. Given the scoping review design, no quantitative meta-analysis was performed. Findings were synthesized descriptively and thematically.

### 2.6. Ethical Considerations

This study followed all ethical guidelines applicable to research that does not involve direct interaction with human or animal participants.

## 3. Results

The database search identified 1726 records. After the removal of 54 duplicate records, 1672 records remained for title and abstract screening. Following screening, 1595 records were excluded, and 77 full-text articles were assessed for eligibility. Of these, 71 articles were excluded, including 45 studies that did not report relevant outcome measures and 26 studies that did not meet the predefined study design criteria. Ultimately, six studies met the inclusion criteria and were included in this scoping review. The study selection process is presented in the PRISMA-ScR flow diagram (Figure 1).

### 3.1. Quality of the Studies

Methodological quality was assessed using study-design-specific appraisal tools. The quasi-experimental study was evaluated using the Joanna Briggs Institute (JBI) Critical Appraisal Checklist, the retrospective cohort study was assessed using the Newcastle–Ottawa Scale (NOS), and RCT were evaluated using the PEDro Scale (Table 1) [40,41,42]. Overall, the included studies demonstrated moderate-to-good methodological quality, with scores ranging from 6/9 on the JBI checklist, 7/9 on the NOS, and from 6/10 to 8/10 on the PEDro Scale (Table 2, Table 3 and Table 4).

### 3.2. Characteristics of the Studies Included

The findings of the six included studies are comprehensively summarized, and their characteristics are presented in Table 5. Most of the scientific articles subjected to in-depth analysis consisted of one experimental group (EG) and one control group (CG). The table outlines the key features of each study, including the author and year of publication, baseline participant characteristics, study design, participant details, intervention protocol, treatment length, outcome measures, and principal findings.

## 4. Discussion

This review synthesizes findings from six studies evaluating PSSE as a standalone treatment, in comparison with other physiotherapeutic approaches, and in combination with adjunct therapies in adolescents and young adults with IS [18,43,44,45,46,47]. The analysis considers participant characteristics, study design, methodological quality, intervention duration, treatment protocols, and structural and postural outcomes, with attention to methodological strengths and limitations.

The studies included a total of 230 participants with mild-to-moderate IS (CA ≤ 40–45°), predominantly female. Samples were generally comparable in age and curve magnitude. Wenxia et al. [18] included 31 participants comparing PSSE with and without MT, Arsovski et al. [43] evaluated PSSE alone in 16 patients, Zhang et al. [45] randomized 42 patients to SE vs. SE + PNF, Abdel-Aziem et al. [47] included 52 patients aged 10–18, Lu et al. [44] assessed 61 patients across SE, SPS, and CSE groups, and Kocaman et al. [46] studied 28 adolescents comparing SE and CSE.

Female participants predominated across studies, consistent with AIS epidemiology. Age range and curve magnitude were similar, indicating representative AIS samples and clinical relevance. Four studies were RCTs [18,45,46,47]. Arsovski et al. [43] conducted an OPSC study without a CG, while Lu et al. [44] used an RCS design with three intervention groups. RCTs provided stronger internal validity, whereas the observational design used by Arsovski et al. [43] was more susceptible to bias due to the absence of a CG. Overall, the included studies demonstrated variable methodological quality, with generally acceptable methodological characteristics but important limitations affecting the certainty of the evidence. RCTs achieved PEDro scores between 6 and 8 out of 10, whereas the retrospective cohort and prospective observational studies scored 7/9 on the NOS and 6/9 on the JBI checklist, respectively. However, the interpretation of these quality assessments should consider the limitations related to small sample sizes, lack of blinding, absence of allocation concealment, and heterogeneity in interventions and outcome measures. Key limitations included small sample sizes, lack of blinding and allocation concealment, and variability in interventions and outcome measures, reducing overall certainty of evidence. Overall, four studies were classified as Level II evidence (RCTs) [18,45,46,47], whereas the remaining studies were classified as Level III evidence according to the National Health and Medical Research Council (NHMRC) and Oxford Centre for Evidence-Based Medicine (OCEBM) criteria [48,49].

Intervention lengths varied considerably across the included studies, limiting direct comparison of outcomes. Wenxia et al. [18], and Abdel-Aziem et al. [47] employed relatively short-term interventions (4 and 10 weeks, respectively), primarily reflecting immediate postural and mobility adaptations. In contrast, Arsovski et al. [43] implemented a longer 3–6-month prospective program, while Zhang et al. [45] delivered 20 supervised sessions over 24 weeks. Similarly, Kocaman et al. [46] applied for 10-week interventions, and Lu et al. [44] conducted a shorter protocol consisting of 10 sessions delivered twice weekly.

All studies used SE-based PSSE as the primary therapeutic approach, while adjunctive interventions varied considerably. Arsovski et al. [43] applied individualized PSSE based on curve type without additional modalities. Zhang et al. [45] added 30 min of PNF-based pelvic rotation correction to each SE session. Wenxia et al. [18] combined 50 min of supervised PSSE with 10 min of MT, three times weekly. However, the contribution of MT to the observed improvements remains uncertain, as evidence is currently limited to a single study and does not allow definitive conclusions regarding whether MT provides additional benefits or influences the long-term effects of exercise-based interventions. Abdel-Aziem et al. [47] combined scoliosis exercises with hippotherapy in the EG. Lu et al. [44] compared SE with SPS exercises and CSE across three groups, while Kocaman et al. [46] directly compared SE and CSE protocols delivered in 45–60-min sessions. Overall, adjunctive interventions targeted specific impairments or functional aspects related to pelvic alignment (PNF), soft-tissue mobility (MT), trunk stabilization (CSE), proprioception and balance (hippotherapy), as well as sensorimotor control with bracing. However, because these modalities were applied in combination with PSSE, their independent effects and specific contribution to the observed outcomes cannot be determined. Therefore, the potential added value of adjunctive interventions requires further investigation through well-designed comparative studies.

The effects of PSSE-based interventions on CA varied across the included studies, depending on the specific intervention protocols, comparison groups, and outcome measures. Wenxia et al. [18] observed a significant CA reduction in the PSSE + MT group (*p* < 0.002), whereas the PSSE-only group showed a slight increase. Abdel-Aziem et al. [47] reported greater reductions in CA and trunk asymmetry when hippotherapy was combined with scoliosis exercises. In contrast, Zhang et al. [45] found only a small, non-significant additional CA reduction (≈1.6°) with PSSE + PNF compared with PSSE alone (*p* = 0.14). Because these adjunctive modalities were applied in combination with PSSE, their independent contribution to the observed changes cannot be determined. Therefore, these findings should be interpreted cautiously, as the limited number and heterogeneity of studies prevent definitive conclusions regarding the superiority of combined or comparative exercise approaches.

Comparative studies evaluating different exercise approaches demonstrated variable findings. Lu et al. [44] reported significant within-group improvements across all interventions (*p* < 0.05). SE and SPS showed greater improvements than CSE in CA and ATR; however, differences were also observed across other postural outcomes, with SE showing greater improvement in C7 plumbline alignment, whereas SPS provided greater benefits for clavicle angle and dynamic postural control. Similarly, Kocaman et al. [46] reported greater improvements with SE than CSE in CA, thoracic ATR, cosmetic trunk deformity, spinal mobility, and QoL (*p* < 0.05), while CSE produced greater gains in peripheral muscle strength, particularly in the knees and upper extremities. These findings suggest potential differences between exercise approaches however, they should be interpreted cautiously due to the limited number of comparative studies, methodological heterogeneity, and differences in intervention protocols and outcome measures. Furthermore, although some studies reported statistically significant reductions in CA, the clinical relevance of these changes remains uncertain, as small variations in CA may fall within the range of measurement error and may not necessarily represent meaningful changes in spinal deformity. Therefore, radiographic findings should be interpreted alongside functional, postural, and patient-centred outcomes.

Beyond radiographic outcomes, several studies reported important sensorimotor and functional benefits of PSSE-based interventions. Zhang et al. [45] reported additional improvements in ATR (*p* = 0.01) with pelvic PNF, while Abdel-Aziem et al. [47] observed better trunk symmetry following hippotherapy. Arsovski et al. [43] also noted improvements in trunk symmetry and rotational deformity, although ATR values were not provided. Overall, adjunctive PSSE interventions appeared to have a greater impact on rotational, proprioceptive, and postural-control outcomes than on CA reduction alone in these short-term studies. Postural and alignment outcomes consistently favoured multimodal physiotherapeutic exercise interventions. All studies reported improvements in pelvic balance, trunk imbalance, and overall posture, with adjunctive interventions generally producing greater effects. Arsovski et al. [43] observed improved pelvic symmetry and trunk balance following individualized PSSE-SE treatment. Zhang et al. [45] reported significantly greater improvements in pelvic obliquity and AVR with PSSE + PNF, while Wenxia et al. [18] found larger reductions in pelvic asymmetry, trunk inclination, and kyphosis with PSSE + MT (*p* < 0.05). Similarly, Abdel-Aziem et al. [47] demonstrated greater decreases in pelvic obliquity, pelvic torsion, and thoracic kyphosis when hippotherapy was combined with SE (*p* < 0.001).

Comparative studies also supported SE-based interventions. Lu et al. [44] showed that SE and SPS were more effective than CSE in reducing CA and ATR (*p* < 0.05), SE significantly improved C7 plumbline alignment, whereas SPS yielded greater improvements in clavicle angle and dynamic postural control. Likewise, Kocaman et al. [46] reported superior outcomes with SE vs. CSE for CA, thoracic ATR, trunk deformity, spinal mobility, and QoL (*p* < 0.05), although CSE was more effective for peripheral muscle strength.

Several methodological strengths enhance the credibility of the included studies. The use of objective and validated instruments including standardized radiographs, Formetric 4D surface topography, inclinometry, Spinal Mouse, Biodex balance system, and validated QoL questionnaires improved measurement accuracy and reduced assessor subjectivity. Four studies employed RCT designs [18,45,46,47], strengthening internal validity and enabling controlled comparisons between PSSE alone, PSSE with adjunctive modalities, and alternative interventions such as CSE and SPS. Additionally, the inclusion of diverse outcome domains structural parameters (CA, ATR, AVR), mobility, balance, postural assessment, QoL, pain, daily functioning, and proprioception provided a comprehensive evaluation of treatment effects. Although two studies used non-RCT or retrospective designs [43,44], they applied the same validated assessment tools and included sensorimotor and postural measures, allowing detailed evaluation of neuromuscular adaptations following PSSE, particularly in balance, postural control, range of motion, trunk endurance, and proprioceptive function.

Clinicians may consider combining PSSE with adjunctive physiotherapeutic approaches to enhance pelvic alignment and neuromuscular control, reflecting the favourable postural outcomes reported in current evidence. However, these findings should be interpreted cautiously until high-quality RCTs with long-term follow-up confirm sustained curve reduction. Schroth-based PSSE may have potential effects on proprioception and sensorimotor control; however, the current evidence is insufficient to establish sensorimotor training as a standard component of scoliosis rehabilitation. Future studies should further investigate the role of sensorimotor approaches in improving postural control, balance, and functional outcomes in individuals with idiopathic scoliosis.

Future research should focus on large, multicentre RCTs using standardized PSSE protocols and clearly defined adjunctive interventions to improve external validity and facilitate comparisons across studies. Longer follow-up periods are needed to assess the durability of treatment effects and clinically relevant outcomes. Dose-response studies should identify the optimal frequency, duration, and intensity of PSSE and adjunct therapies to establish evidence-based treatment guidelines. Additionally, future trials should incorporate blinded outcome assessment, preregistered protocols, and rigorous methodological controls to reduce bias and enhance the reliability and reproducibility of findings. Moreover, future investigations should seek to determine which subgroups of patients with IS are most likely to benefit from specific combinations of PSSE and adjunctive interventions, thereby supporting a more individualized and precision-based rehabilitation approach.

Despite positive trends, the evidence has important limitations that affect the generalizability and interpretation of the findings. One of the major limitations of this review is the considerable heterogeneity among the included studies with respect to participants’ age, scoliosis severity, intervention protocols, treatment duration, adjunctive interventions, and outcome measures. Most studies had small sample sizes (*n* = 16–61), reducing statistical power and increasing the risk of Type II error, particularly when detecting modest changes in CA. Limited blinding, non-randomized designs [43,44], and the absence of assessor blinding in several studies further increased the risk of selection, performance, and detection bias. Furthermore, the inclusion of multimodal interventions combining PSSE with MT, PNF, hippotherapy, or other therapeutic approaches limits the ability to determine whether the reported improvements are attributable primarily to PSSE or to the combined treatment effects. Comparative studies also varied in the type and structure of alternative interventions (e.g., CSE, SPS), making it difficult to determine whether the superiority of SE-based approaches resulted from specific exercise principles, treatment intensity, therapist supervision, or adherence. The lack of standardized comparative protocols further restricts cross-study comparisons. Furthermore, PSSE should not be interpreted as a single homogeneous intervention, as it represents a group of exercise approaches based on different theoretical principles and therapeutic strategies. Therefore, variations in exercise concepts, treatment dosage, therapist expertise, and adjunctive components may have influenced the observed outcomes. Direct comparisons between different PSSE approaches are needed to determine whether specific methods provide superior effects on structural and secondary postural outcomes.

Follow-up periods were generally short (≤6 months), limiting conclusions regarding long-term curve progression and clinically important outcomes such as bracing or surgery. Wenxia et al. [18] highlighted the need for rigorous RCTs with standardized PSSE protocols, adequate sample sizes, and long-term (≥1 year) follow-up to confirm these preliminary findings. Current evidence suggests that adding targeted modalities such as pelvic PNF, MT, and hippotherapy to SE-based PSSE may enhance short-term improvements in ATR and posture, although changes in CA remain modest. Comparative studies further indicate that SE-based PSSE may be more effective than CSE and SPS in improving primary and secondary postural and functional outcomes. The inclusion of sensorimotor measures also expands the understanding of PSSE effects beyond radiographic correction, demonstrating potential benefits in neuromuscular organization, balance, and proprioception. Collectively, these findings support the need for integrative biomechanical and neurophysiological models to better explain how secondary postural adaptations respond to targeted physiotherapeutic interventions. Consequently, the mechanisms through which PSSE influences secondary postural adaptations remain incompletely understood, and further research integrating biomechanical, neuromuscular, and sensorimotor frameworks is warranted.

Furthermore, PSSE should not be interpreted as a single homogeneous intervention, as it represents a group of exercise approaches based on different theoretical principles and therapeutic strategies. Therefore, variations in exercise concepts, treatment dosage, therapist expertise, and adjunctive components may have influenced the observed outcomes. Direct comparisons between different PSSE approaches are needed to determine whether specific methods provide superior effects on structural and secondary postural outcomes. Moreover, the sustainability of treatment effects remains unclear, as most included studies assessed short-term outcomes with follow-up periods of six months or less. Future research should include longer follow-up periods to determine whether improvements in spinal parameters, posture, and functional outcomes are maintained over time.

### Practical Implications

The findings have important clinical implications for healthcare professionals, particularly physiotherapists managing IS. A structured, conservative approach incorporating PSSE, especially when combined with adjunctive therapies, may be considered for children, adolescents, and young adults with mild to moderate IS. Treatment should include individualized exercise programs, appropriate adjunctive interventions, and regular monitoring, while recognizing the limited high-quality evidence on long-term outcomes. Comparative evidence suggests that SE-based PSSE may be more effective than CSE in improving spinal alignment, trunk rotation, postural symmetry, spinal mobility, and QoL. However, SPS may offer greater benefits in dynamic postural control and functional performance, suggesting that different exercise modalities target distinct functional domains. Therefore, exercise selection should be individualized based on postural deficits, sensorimotor impairments, and rehabilitation goals. The findings also support integrating sensorimotor and postural-control training into scoliosis rehabilitation, as improvements in balance, proprioception, and neuromuscular control may enhance functional and postural adaptation beyond structural correction alone. Accordingly, rehabilitation goals should extend beyond radiographic correction and incorporate patient-reported outcomes, movement quality, functional performance, and participation in everyday life.

## 5. Conclusions

This scoping review suggests that PSSE may contribute to improvements in structural parameters and secondary postural deviations in individuals with IS. Although PSSE alone demonstrated potential benefits, some studies reported greater improvements when PSSE was combined with adjunctive physiotherapeutic interventions targeting postural alignment, sensorimotor control, and functional outcomes. Comparative evidence suggests potential differences between SE-based PSSE and CSE for selected structural and postural outcomes; however, these findings remain limited by the heterogeneity and small number of comparative studies. Overall, current evidence highlights the importance of considering biomechanical, functional, and patient-centred outcomes alongside structural parameters. Sensorimotor aspects may represent a promising area for future rehabilitation research; however, further high-quality studies are required before specific recommendations can be established. The available evidence suggests that PSSE may have beneficial effects beyond radiographic parameters, particularly regarding postural control and functional outcomes. However, further high-quality RCTs with standardized protocols and longer follow-up periods are required to clarify the independent and combined effects of PSSE and adjunctive interventions.

## Figures and Tables

**Table 1:** Quality assessment summary.

Article	Study Design	Assessment Tool	Score	Quality
Arsovski et al. [43]	OPSC	JBI	6/9	Moderate
Lu et al. [44]	RCS	NOS	7/9	Good
Zhang et al. [45]	RCT	PEDro	8/10	Good
Wenxia et al. [18]	RCT	PEDro	6/10	Good
Kocaman et al. [46]	RCT	PEDro	8/10	Good
Abdel-Aziem et al. [47]	RCT	PEDro	7/10	Good

OPSC: Observational, prospective, single-centre; RCS: Retrospective cohort study; RCT: Randomized controlled trial; JBI: Joanna Briggs Institute Critical Appraisal Checklist; NOS: Newcastle–Ottawa Scale; PEDro: Physiotherapy Evidence Database.

**Table 2:** Quality assessment using the JBI critical appraisal checklist.

Study	Q1	Q2	Q3	Q4	Q5	Q6	Q7	Q8	Q9	Total Score	Quality Rating
Arsovski et al. [43]	Y	N	N	N	Y	Y	Y	Y	Y	6/9	Moderate

JBI: Joanna Briggs Institute Critical Appraisal Checklist; Scoring: N: criterion not fulfilled; Y: criterion fulfilled; Y = 1 point; N = 0 points; maximum total = 9 points; JBI Criteria: Q1 = Clear cause-and-effect relationship; Q2 = Similarity of participants included in comparisons; Q3 = Similarity of treatment/care other than the intervention of interest; Q4 = Presence of a control group; Q5 = Multiple measurements before and after intervention; Q6 = Complete follow-up or adequate handling of loss to follow-up; Q7 = Reliable outcome measurement; Q8 = Appropriate statistical analysis; Q9 = Consistent intervention delivery. Quality classification: Poor (0–3), Moderate (4–6), Good (7–9).

**Table 3:** Quality assessment using the NOS.

Study	S1	S2	S3	S4	C1	C2	O1	O2	Total
Lu et al. [44]	Y	Y	Y	Y	N	N	Y	Y	7/9

NOS: Newcastle–Ottawa Scale; Scoring: N: criterion not fulfilled; Y: criterion fulfilled; Y = 1 point; N = 0 points; maximum total = 9 points; S1 = Representativeness of the exposed cohort; S2 = Selection of the non-exposed cohort; S3 = Ascertainment of exposure; S4 = Demonstration that the outcome of interest was not present at the start of the study; C1 = Comparability of cohorts on the basis of design or analysis (primary factor); C2 = Comparability of cohorts on the basis of design or analysis (additional factor); O1 = Assessment of outcome; O2 = Was follow-up long enough for outcomes to occur; O3 = Adequacy of follow-up of cohorts. Higher scores indicate better methodological quality.

**Table 4:** Quality assessment using the PEDro scale.

	Items by Number on the PEDro Scale												
Article	1	2	3	4	5	6	7	8	9	10	11	=	Quality
Zhang et al. [45]	Y	Y	Y	N	N	Y	Y	Y	Y	Y	Y	8	Good
Wenxia et al. [18]	Y	N	N	Y	N	N	Y	Y	Y	Y	Y	6	Good
Kocaman et al. [46]	Y	Y	Y	Y	N	N	Y	Y	Y	Y	Y	8	Good
Abdel-Aziem et al. [47]	N	Y	Y	Y	N	N	Y	Y	N	Y	Y	7	Good

PEDro: Physiotherapy Evidence Database; N: criterion not fulfilled; Y: criterion fulfilled; 1: eligibility criteria were specified; 2: subjects were randomly allocated to groups or to a treatment order; 3: allocation was concealed; 4: the groups were similar at baseline; 5: all subjects were blinded; 6: all therapists were blinded; 7: all assessors were blinded; 8: measures of at least one key outcome were obtained from over 85% of the subjects who were initially allocated to groups; 9: intention-to-treat analysis was performed on all subjects who received the treatment or control condition as allocated; 10: the results of between-group statistical comparisons are reported for at least one key outcome; 11: the study provides both point measures and measures of variability for at least one key outcome. Each satisfied item (except the first) contributes 1 point to the total score, yielding a PEDro scale score that can range from 0 to 10. The PEDro scale categorizes methodological rigor into four levels: excellent (9–10), good (6–8), fair (4–5), and poor (<4).

**Table 5:** Summary of included studies in the review.

Author and Year	Baseline Characteristics	Study Design Participants	Treatment Length	Intervention Details	Outcomes Measured	Results
Arsovski et al. [43]	N = 16, 12 female, four male with IS. Mean age 18.6 ± 12.4 years. Majority adolescents (*n* = 13; 81.25%). Minority of slightly older participants (*n* = 3; 18.75%) CA ≤ 40°	OPSC—Observational, Prospective, Single-Centre	3–6 months two to three sessions per week, 45–60 min each	Individualized PSSE–SE program tailored to curvature type (3C+, N3N4, 4C), supervised by a certified therapist	Structural (CA, Risser sign) Clinical postural assessment (photographic postural analysis, pelvic imbalance) Functional mobility (ROM, trunk endurance, postural control) QoL (pain, daily function)	Majority of participants (81.25%) showed improvements in postural symmetry, pelvic alignment, trunk control, and functional stability, with a clinically meaningful reduction in CA and ATR. No significant age or gender differences were found in pelvic alignment (*p* > 0.05). A borderline association was observed between Risser grade and scoliosis type (*p* = 0.057).
Lu et al. [44]	N = 61 adolescents with idiopathic scoliosis (IS), 50 female, 11 male. Mean age aged 6–18 years, Cobb angle ≤40°.	RCS within three groups: SE (*n* = 22), SPS (*n* = 21), and CSE (*n* = 18).	10 sessions.	SE, PSSE, and CSE were compared across three groups, with all groups performing 30-min exercise sessions 2×/week.	CA, ATR, trunk aesthetics (TRACE), pain (VAS), radiographic alignment (C7PL, clavicle angle, pelvic obliquity), QoL (SRS-22)	The findings revealed significant within-group improvements in CA, pain, and ATR for all interventions (*p* < 0.05). Both SE and SPS achieved greater reductions in CA (*p* = 0.016; *p* = 0.033) and ATR (*p* = 0.001; *p* = 0.008) than CSE. SPS showed superior improvement in clavicle angle and postural control (*p* < 0.001), while SE significantly improved C7PL alignment (*p* = 0.030).
Zhang et al. [45]	N = 42, adolescents aged 14–16 years. Mean age 13.33 ± 2.41 years. Mild AIS (CA < 25°)	RCT with two groups: SE (*n* = 21) SE + PNF (*n* = 21)	24 weeks (20 sessions)	CG: SE EG: SE + PNF: Schroth + pelvic rotation correction program based on PNF	Primary outcome: Concave/convex hip bone width ratio Secondary outcome: CA, ATR SRS-22 self- image AVT, AVR Pelvic obliquity	The findings demonstrated significantly great improvements in concave/convex ratio (*p* < 0.001), ATR (*p* = 0.01), and AVR (*p* = 0.04) in the EG (SE + PNF) compared with the CG (SE). Improvements in self-image measured by SRS-22 also favoured the EG (SE + PNF), reaching borderline statistical significance (*p* = 0.049). However, no significant between-group differences were observed for CA (*p* = 0.14), AVT (*p* = 0.72), or pelvic obliquity (*p* = 0.52).
Wenxia et al. [18]	N = 31, adolescents mean age: EC = 12.53(1.32), CG = 13.43 (2.27) AIS (CA 10–45°).	RCT with two groups. EG: PSSE + MT (*n* = 17) CG: PSSE (*n* = 14)	4 weeks (three sessions/week)	EG: 50 min PSSE + 10 min MT per session; CG: 50 min PSSE (as their home exercise program).	CA Spinal mobility Trunk shape with DIERS Formetric4D (Trunk length, sagittal imbalance, coronal imbalance, kyphotic angle, lordotic angle, vertebral rotation (RMS/MAX), apical deviation (RMS/MAX), pelvic obliquity, pelvic torsion), movement capability, and QOL	The findings demonstrated significant differences favouring the EG (PSSE + MT), in Functional Movement Screen (FMS), SRS-22 total score, and several spinal range of motion (ROM) parameters, including thoracic flexion and lumbar flexion/extension and lateral flexion (all *p* < 0.01). Significant improvements were also observed in trunk morphology indices, including vertebral rotation (RMS/MAX), apical deviation, and pelvic obliquity (*p* < 0.05). No significant differences were found for coronal and sagittal imbalance or lordotic and kyphotic angles (*p* > 0.05). According to MCID analysis, the intervention group achieved clinically meaningful improvements across most ROM and trunk shape parameters, whereas the CG did not.
Kocaman et al. [46]	N = 28 adolescents with AIS (6–18 years) CA 10–26°)	Single-blind RCT EG: SE (*n* = 14) CG: CSE (*n* = 14)	10 weeks, three sessions/ week, and both groups were additionally prescribed traditional exercises to perform.	SE and CSE 45–60 min per session (both groups)	Primary outcome: CA (radiography). Secondary outcome: ATR (Adam’s test), cosmetic trunk deformity (WRVAS), spinal mobility (Spinal Mouse), peripheral muscle strength (Biodex System 4-Pro), and QoL (SRS-22)	The results show that both exercise programs significantly improved outcomes; however, the EG (SE) achieved greater improvements in spinal deformity, mobility, cosmetic appearance, and QoL. Specifically, EG had larger reductions in CA, ATR-T, and WRVAS scores, as well as greater gains in spinal mobility and SRS-22 compared with the CG (CSE) (*p* < 0.05). In contrast, the CG showed superior improvements in peripheral muscle strength of both the upper and lower extremities (*p* < 0.05). Overall, the EG (SE) were more effective for spinal correction and functional outcomes, while CG (CSE) were more effective for peripheral muscle strength.
-Abdel-Aziem et al. [47]	N = 52, adolescents aged 10–18. AIS (CA 18–19°)	RCT—Randomized into two matched groups by CA and age: EG *n* = 27 (19 female/eight male), aged 14.74 ± 1.79 years; CA 18.59 ± 2.66 degrees CG: *n* = 25 (18 female/seven male), aged 15.04 ± 1.81 years; CA 19.32 ± 2.69 degrees	10 weeks, three sessions per week.	EG: SE + hippotherapy; CG: SE only. Each session: 45–60 min	Scoliotic angle (CA) Kyphotic angle Pelvic obliquity Pelvic torsion Vertical spinal rotation Balance indices (anteroposterior, mediolateral and overall stability)	The scoliotic angle improved in both groups, but more in the EG (SE + hippotherapy) (24.09° → 18.41°) than in the CG (SE) (25.06° → 22.32°) (*p* < 0.001). The kyphotic angle also decreased in the EG (49.26° → 44.26°) compared with the CG (50.32° → 48.00°) (*p* < 0.001). Pelvic obliquity improved in both groups (EG: 4.91° → 2.37°; CG: 4.99° → 3.08%; *p* < 0.05). Overall, the EG showed significantly greater improvements in postural asymmetry and balance compared with the control group (*p* < 0.001).

AIS—adolescent idiopathic scoliosis; IS—idiopathic scoliosis; *n*—sample size; EG—experimental group; CG—control group; CA—Cobb angle; ATR—angle of trunk rotation; AVT—apical vertebral translation; AVR—apical vertebral rotation; SE—Schroth exercises; CSE—core stabilization exercises; SPS—Spiral Stabilization; MT—manual therapy; PNF—proprioceptive neuromuscular facilitation; SE + PNF—Schroth exercises combined with pelvic rotation correction based on PNF; QoL—quality of life; FMS—Functional Movement Screen; WRVAS—Walter Reed Visual Assessment Scale; TRACE—Trunk Aesthetic Clinical Evaluation; VAS—Visual Analog Scale; C7PL—C7 plumb line alignment; RCT—randomized controlled trial; RCS—retrospective cohort study; OPSC—observational prospective single-centre study; Spinal Mouse—spinal mobility assessment system.

## Data Availability

The original data and materials presented in this study are included within the article and its Supplementary Materials. Additional inquiries may be directed to the corresponding author.

## Supplementary Materials

The following supporting information can be downloaded at: https://www.mdpi.com/article/10.3390/jfmk11030288/s1, Supplementary File S1: Complete search strategies and retrieved search results.

## Abbreviations

The following abbreviations are used in this manuscript:

ADLs	Activities of daily living
ATR	Angle of trunk rotation
AVR	Apical vertebral rotation
AVT	Apical vertebral translation
C7PL—C7	Plumb line alignment
CCT	Controlled clinical trial
CG	Control group
CA	Cobb angle
CSE	Core stabilization exercises
EG	Experimental group
FMS	Functional Movement Screen
FITS	Functional Individual Therapy for Scoliosis
IS	Idiopathic scoliosis
JBI	Joanna Briggs Institute
MT	Manual therapy
NHMRC	National Health and Medical Research Council
NOS	Newcastle–Ottawa Scale
OPSC	Observational prospective single-centre study
OCEBM	Oxford Centre for Evidence-Based Medicine
PEDro	Physiotherapy Evidence Database Scale
PSSE	Physiotherapy Scoliosis-Specific Exercises
PCC	Population, Concept, and Context framework
PRISMA-ScR	Preferred Reporting Items for Systematic Reviews and Meta-Analyses Extension for Systematic Reviews
PNF	Proprioceptive Neuromuscular Facilitation
QoL	Quality of life
RCT	Randomized controlled trials
RCS	Retrospective cohort study;
ROM	Range of motion
SE	Schroth exercise
SEAS	Scientific Exercise Approach to Scoliosis
SOSORT	Scoliosis Orthopaedic and Rehabilitation Treatment
SPS	Spiral stabilization
TRACE	Trunk Aesthetic Clinical Evaluation
VAS	Visual Analog Scale
WRVAS	Walter Reed Visual Assessment Scale
