# Supplementary material for: The Evidence of Physiotherapy Scoliosis-Specific Exercises in Treating Secondary Postural Deviations in Mild and Moderate Idiopathic Scoliosis: A Scoping Review
Source: J Funct Morphol Kinesiol. 2026 Jul 24;11(3):288. doi: 10.3390/jfmk11030288 (PMC13510499; doi:10.3390/jfmk11030288)
Supplement: Supplementary file 1 [file jfmk-11-00288-s001.zip › jfmk-4418385-File S1.pdf]

Search strategy

1. PubMed
“Adolescent Idiopathic Scoliosis” [mh] 2. “Adolescent Idiopathic Scoliosis” [tw] 3. AIS [tw] 4. “Idiopathic scoliosis” [tw] 5. “Mild and moderate idiopathic scoliosis” [tw] 6. OR/1–5 7. PSSE [tw] 8. “Physiotherapy scoliosis-specific exercises” [tw] 9. “Physiotherapeutic exercises” [tw] 10. OR/7–9 11. Child[mh] 12. Adolescent[mh] 13. Adult[mh] 14. Child*[tw] 15. Adolescen*[tw] 16. Adult*[tw] 17. OR/11–16 18. “Pelvic asymmetry” [tw] 19. “Pelvic position” [tw] 20. OR/18–19 21. “Trunk asymmetry” [tw] 22. “Trunk imbalance” [tw] 23. “Postural deviation*” [tw] 24. OR/21–23 25. “Posture correction” [tw] 26. “Asymmetry correction” [tw] 27. OR/25–26 28. “Sagittal curvature” [tw] 29. “Secondary postural deviation*” [tw] 30. OR/28–29 31. (6 AND 10 AND 17 AND 20 AND 24 AND 27 AND 30) AND humans[mh]

2. PEDro

Adolescent Idiopathic Scoliosis OR AIS OR idiopathic scoliosis OR mild and moderate idiopathic scoliosis AND PSSE OR physiotherapy scoliosis-specific exercises OR physiotherapeutic exercises AND child OR adolescent OR adult* AND pelvic asymmetry OR pelvic position AND trunk asymmetry OR trunk imbalance OR postural deviation* AND posture correction OR asymmetry correction AND sagittal curvature OR secondary postural deviation*

3. Google Scholar

“Adolescent Idiopathic Scoliosis” OR AIS OR “idiopathic scoliosis” OR “mild and moderate idiopathic scoliosis” AND “PSSE” OR “physiotherapy scoliosis-specific exercises” OR “physiotherapeutic exercises” AND child OR adolescent OR adult* AND “pelvic asymmetry” OR “pelvic position” AND “trunk asymmetry” OR “trunk imbalance” OR “postural deviation*” AND “posture correction” OR “asymmetry correction” AND “sagittal curvature” OR “secondary postural deviation*”

4. Scopus

TITLE-ABS-KEY (“Adolescent Idiopathic Scoliosis” OR AIS OR “idiopathic scoliosis” OR “mild and moderate idiopathic scoliosis”) AND TITLE-ABS-KEY (“PSSE” OR “physiotherapy scoliosis-specific exercises” OR “physiotherapeutic exercises”) AND TITLE-ABS-KEY (“child” OR “adolescent” OR “adult*”) AND TITLE-ABS-KEY (“pelvic asymmetry” OR “pelvic position”) AND TITLE-ABS-KEY (“trunk asymmetry” OR “trunk imbalance” OR “postural deviation*”) AND TITLE-ABS-KEY (“posture correction” OR “asymmetry correction”) AND TITLE-ABS-KEY (“sagittal curvature” OR “secondary postural deviation*”)
